# Less than one-fifth of the mothers practised exclusive breastfeeding in the emerging regions of Ethiopia: a multilevel analysis of the 2016 Ethiopian demographic and health survey

**DOI:** 10.1186/s12889-020-10071-2

**Published:** 2021-01-04

**Authors:** Tsegaye Gebremedhin, Demiss Mulatu Geberu, Asmamaw Atnafu

**Affiliations:** Department of Health Systems and Policy, Institute of Public health, College of Medicine and Health Sciences, University of Gondar, P. O. Box: 196, Gondar, Ethiopia

**Keywords:** Exclusive breastfeeding, Pastoral communities, Multilevel analysis, Ethiopia

## Abstract

**Background:**

The burden of low coverage of exclusive breastfeeding (EBF) has a significant impact on the health of a newborn and also on the family and social economy in the long term. Even though the prevalence of EBF practices in Ethiopia is low, the practices in the pastoral communities, in particular, are significantly low and affected by individual and community-level factors. Besides, its adverse outcomes are mostly unrecognised. Therefore, this study aimed to assess the individual and community-level factors of low coverage of EBF practices in the emerging regions of Ethiopia.

**Methods:**

In this analysis, data from 2016 Ethiopian Demographic and Health Survey (EDHS) were used. A two-stage stratified sampling technique was used to identify 1406 children aged 0 to 23 months in the emerging regions of Ethiopia. A multilevel mixed-effect binary logistic regression analysis was used to determine the individual and community level factors associated with exclusive breastfeeding practices. In the final model, variables with a *p*-value of < 0.05 and Adjusted Odds Ratio (AOR) with 95% Confidence Interval (CI) were found to be statistically significant factors that affect exclusive breastfeeding practices.

**Results:**

Overall, 17.6% (95% CI: 15.6–19.6) of the children aged 0 to 23 months have received exclusive breastfeeding. Employed mothers (AOR: 0.33, 95% CI: 0.21–0.53), richer household wealth status (AOR: 0.39, 95% CI: 0.16–0.96), mothers undecided to have more children (AOR: 2.29, 95% CI: 1.21–4.29), a child with a history of diarrhoea (AOR: 0.31, 95% CI: 0.16–0.61) were the individual-level factors, whereas Benishangul region (AOR: 2.63, 95% CI: 1.44–4.82) was the community-level factors associated with the exclusive breastfeeding practices.

**Conclusions:**

Less than one-fifth of the mothers have practised exclusive breastfeeding in the emerging regions of Ethiopia. The individual-level factors such as mother’s employment status, household wealth status, desire for more children, presence of diarrhoea and community-level factors such as region have contributed to the low coverage of exclusive breastfeeding. Therefore, the federal and regional health bureaus and other implementers should emphasise to those emerging regions by creating awareness and strengthening the existing community-based health extension program to enhance exclusive breastfeeding practices.

## Background

The World Health Organization (WHO) define Exclusive Breastfeeding (EBF) as the condition in which an infant receives only breast milk from his/her mother or a wet nurse and no other liquids or solids except drugs or syrups composed of vitamins, mineral supplements or drugs [1]. Exclusive breastfeeding improves the healthy development of infants and protects against the common childhood illness [2–6]. Similarly, breastfeeding is essential to the baby’s health, which strengthens the physical and spiritual bond between mothers and their children [7]. It also provides infants with superior nutritional content that is capable of enhancing infant immunity and possible reduction in future health care spending [8, 9]. Improvements in rates of exclusive breastfeeding can avert a large number of child death, disease burden, and child mortality at an individual level and inequalities in developing countries at large [10]. Moreover, it is a proven effective child health intervention that does not require extensive health-system infrastructure [11–17].

Even though the benefits of exclusive breastfeeding for newborns and young infants up to the age of 6 months are well proven, globally, the majority of the mothers practised it sub-optimally [18]. The size of the gap between the current practice and recommendations is large when one considers breastfeeding involves no out-of-pocket costs, presence of universal consensus on best practices, and that implementing current recommendations could save the life of 1.45 million children each year in developing countries [4].

Exclusive breastfeeding practices ranged from 3.5% in Djibouti to nearly 80% in Rwanda in 2010 [10]. In Ethiopia, the national prevalence of EBF was 59.9% [19, 20] and in the agrarian communities, it ranges from 29.3% in Addis Ababa [21] to 79% in Azezo district, northwest Ethiopia [22–24]. Besides, in the pastoral communities of Ethiopia, the prevalence of exclusive breastfeeding ranges from 24.8% in Fafan zone to 55.0% in Asayita woreda [25–27].

Despite its benefits, exclusive breastfeeding practices can be adversely affected by factors such as political, socio-economic, cultural, and individual factors related to both mother and baby [28, 29]. The reviewed literature identified only the individual and household level factors that affect the exclusive breastfeeding practices, particularly in resource-limited settings, but the community-level factors might significantly affect the EBF practices.

Some of the documented factors associated with failure to exclusively breastfeeding includes residence, marital status, types of occupation, accessibility of health facilities, knowledge of mothers about exclusive breastfeeding, maternal health problem, and low utilisation of maternal health services [30–34]. EBF is affected by factors such as the perception that babies continued to be hungry after breastfeeding, maternal health problems, fear of babies becoming addicted to breast milk, pressure from the mother-in-law, pains in the breast, and the need to return to work [7]. Additionally, marital status, accessibility of health facility and knowledge about child feeding practice can affect the breastfeeding practices [35].

Child characteristics such as sex, birth order, the season of birth, and age may also affect breastfeeding practices. A study conducted in Burao district, Somaliland, showed that the exclusive breastfeeding practices were lower among female children [36]. Likewise, higher birth order might increase the burden for the mother and subsequently affects exclusive breastfeeding practices [37]. Moreover, studies conducted in Jigjiga showed that EBF was more likely practised by mothers who had infants aged 0–1 year, mothers who did not feed their current infant from bottles on the proceeding day, and mothers who did not feed infant formula to their previous infants [26]. A decline in breastfeeding is associated with women involvement in the workforce, lack of knowledge on the benefits of the practice and management of lactation problems [38].

Empirical evidence showed that the burden of low coverage of exclusive breastfeeding has a great impact on the health of a newborn and economic impact on its family. Suboptimal and ephemeral breastfeeding is also associated with maternal adverse health outcomes and family economic costs [39, 40]. Suboptimal breastfeeding is a leading childhood risk factor in all developing countries and consistently ranks higher than water and sanitation [41]. Suboptimal breastfeeding practices accounted for 56.5, 39.0, and 22.8% of diarrhoea deaths in the late neonatal, infancy period, and children aged under 5 years, respectively, in Nigeria [42].

A study conducted in Mexico showed that maternal morbidity, premature death, and economic costs covered by health sectors and society for the treatment of breast cancer due to suboptimal breastfeeding practices is high [43]. A study conducted in the US showed that suboptimal breastfeeding could increase maternal morbidity and health care costs; suboptimal breastfeeding incurs a total of $17.4 billion to US society resulting from premature death [40].

The prevalence of EBF practices in the pastoral communities of Ethiopia is significantly below the national and WHO recommendations. Besides, the health, as well as economic costs of suboptimal breastfeeding, are largely unrecognised. Moreover, considering the high percentage of suboptimal infant feeding practices in those regions characterised by poor infrastructure, inaccessibility of health services, drought, and poverty the negative consequence of the suboptimal breastfeeding practice might be significant. Therefore, this study aimed to assess the individual and community level factors of low coverage of exclusive breastfeeding practices among children aged 0–23 months in the emerging regions of Ethiopia based on 2016 Ethiopian demographic and health survey (EDHS).

## Methods

### Study settings and data source

The 2016 Ethiopian Demographic and Health Survey data were used for this study. The EDHS is a nationally representative household survey data that has been implemented by the Central Statistical Agency (CSA) of Ethiopia, every 5 years [20]. Ethiopia is divided into two administrative cities and nine regions. These regions are again categorised underdeveloped and emerging regions. The emerging regions are Afar, Somali, Benishangul, and Gambela, where scattered pastoralists predominantly live. Moreover, inadequate infrastructure, inaccessibility of health services, drought, poverty and absence of clear as well as detailed regulations are their common characteristics in emerging regions [44, 45]. Whereas, the developed regions are Amhara, Oromia, Tigray, South Nation Nationalities and Peoples’ Region (SNNPR) and Harari regions and the city administrations characterised by a relatively denser population and better infrastructure, access to health and education.

### Sampling procedures

The 2016 EDHS was used the Ethiopian population and housing census, which was conducted in 2007 by the Ethiopian CSA, as a sampling frame. The census used a complete list of 84,915 enumeration areas (EA) created for the 2007 Primary Health Care (PHC) as a frame. The sampling frame contains the EA, location, type of residence, and the estimated number of residential households. The 2016 EDHS was stratified in two stages, and samples of EA were selected independently in each stratum.

In this study, the 2016 Ethiopian demographic and health survey childhood datasets of the four emerging regional states, namely Afar, Benishangul, Gambella, and Somali, were used for analysis.

All women aged 15–49 years who are the regular members of the selected households were eligible for the female survey. Children aged 0–23 months are the study population. Those non-alive and live with other than their mothers were excluded from the analysis. Finally, a total of 1406 mothers with their children aged 0–23 months were included in the analysis, and data on both were extracted from the 2016 EDHS datasets using STATA version 14 software. Potential individual and community level independent variables were also extracted, and further analysis of the selected variables was done.

### Measurements of variables

Exclusive breastfeeding practices was the dependent variable which was measured in two ways for the age groups of less than 6 months and 6–23 months. For mothers who had less than 6 months old children during the data collection period were asked about the feeding of breast milk without anything else in the last 24 h preceding the survey, except for Oral Rehydration Salt (ORS), syrups (vitamins, minerals, medicines), and others for therapeutic purposes. Whereas, mothers who had 6 to 23 months old children were asked about their lifelong (about 6 months) EBF practices using since birth dietary recall method retrospectively [24, 36, 46]. The information on exclusive breastfeeding was collected from mothers’ verbal responses. The mothers were asked about their children current breastfeeding status, the timing of breastfeeding initiation and exclusive breastfeeding practices. Exclusive breastfeeding for infants should be practised for the first 6 months, and then for 18 additional months and more along with complementary foods for better health and development as per the world health organisation recommendation’. On 18 May 2001, the world health assembly urged the member states to promote EBF for 6 months as a global public health recommendation [47, 48].

Two sets of explanatory variables (individual and community–level) were included in this study. Both maternal (socio-demographic and maternal health service-related characteristics) and child-related variables were included in the individual level-variables. Whereas, place of residence, region, distance to a health facility, community-level poverty, and media exposure were the community-level variables.

Distance to a health facility was assessed by the question “distance to the nearest health facility is a problem?” and the responses were categorised as “big problem” or “not a problem”.

Women empowerment was assessed using decision making power and justification of wife-beating. Women who were empowered were those who participated in decision making either alone or jointly with their husbands in all instances and did not ever justify wife-beating.

Community-level poverty was assessed using the asset index based on data from the entire sample on separate scores prepared for rural and urban households, and combined to produce a single asset index for all households as community level and ranked into three (poor, middle, and rich).

Community media exposure was assessed as “yes” if they have access to all three media (newsletter, radio, and TV) at least once a week, otherwise “no”.

### Data processing and statistical analysis

The data were extracted, cleaned, re-coded, and analysed using STATA version 14 (Stata Corp, College Station, TX). Descriptive statistics were presented using tables and narrations. A multilevel mixed-effect logistic regression analysis was conducted after checking the eligibility. The model eligibility was assessed by calculating the Intra-class Correlation Coefficient (ICC) (ICC greater than 5% is eligible for multilevel analysis). In our study, the ICC was 45.0%. Since the DHS data are hierarchical (individual were nested within communities), a two-level mixed-effects logistic regression model was fitted to estimate both the individual and community level variables (fixed and random) effects on exclusive breastfeeding practices [49];

Bi-variable and multivariable analysis were computed. First, in the bi-variable logistic regression analysis, a *p*-value of less than 0.2 was used to fit the three models (model 1: individual level, model 2: community level, and model 3: both the individual and community level). In the final model (model 3) (mixed-effect), a *p*-value less than 0.05 and adjusted odds ratio (AOR) with 95% confidence interval (CI) were used to declare statistically significant factors with the exclusive breastfeeding practice among children aged 0 to 23 months of children in the emerging regions of Ethiopia.

The measures of variation (random-effects) between clusters were reported using ICC. The ICC refers to the ratio of the between-cluster variance to the total variance, and it tells us the proportion of the total variance in the outcome variable that is accounted at the cluster level. Akaike’s information criterion (AIC) was used to estimate the goodness of fit of the adjusted final model in comparison with the preceding models.

## Results

### Socio-demographic and economic characteristics of participants

A total of 1406 mothers/caregivers with their children aged 0–23 months were included in this analysis. The mean age of the mothers was 27.37 ± 6.19 years, and the majority (84.2%) lives in rural area. Nearly 96 % of the women were married, and 71.9% were Muslim. Seventy percent of the women and 57.8% of their husbands/partners had no education. Moreover, 69.2% of the women had no work and 61.9% were in the poorest wealth status. The mean family size was 6 ± 2.4 (Table 1).

**Table 1:** Socio-demographic and economic characteristics of study participants in emerging regions of Ethiopia, EDHS 2016 (*n* = 1406)

Variables	Category	Frequency (n)	Percent (%)
Age of mothers/caretakers in complete years	15–24	487	34.7
	25–30	557	39.6
	31–35	217	15.4
	> = 36	145	10.3
Residence	Urban	222	15.8
	Rural	1184	84.2
Region	Afar	358	25.5
	Somali	496	35.3
	Benishangul_Gumuz	307	21.8
	Gambella	245	17.4
Religion	Muslim	1011	71.9
	Protestant	248	17.6
	Orthodox	109	7.8
	Others	38	2.7
Sex of head of household	Male	952	67.7
	Female	454	32.3
Current marital status	Married	1347	95.8
	Unmarried	59	4.2
Educational status of mothers/caretakers	No education	985	70.0
	Primary education	286	20.4
	Secondary education	99	7.0
	Diploma and above	36	2.6
Educational status of husband’s/partner’s (*n* = 1347)	No education	778	57.8
	Primary education	286	21.2
	Secondary education	138	10.2
	Higher	131	9.7
	Do not know	14	1.1
Respondent’s occupation	No work	973	69.2
	Technical	106	7.5
	Agricultural	236	16.8
	Others	91	6.5
Husbands’ occupation (*n* = 1347)	No work	200	14.9
	Technical	256	19.0
	Agricultural	619	46.0
	Others	272	20.1
Wealth status	Poorest	870	61.9
	Poorer	159	11.3
	Middle	101	7.2
	Richer	100	7.1
	Richest	176	12.5
Family size	< 6	640	45.5
	6+	766	54.5

### Obstetric history of participants

The majority (56.0%) of the women had antenatal care (ANC) visits for their recent pregnancy. Of those who had ANC visits, 44.3% had four and more times visits, and only 10% visited before the third month for their first antenatal care. Nearly 90 % of the women wanted their pregnancy, and 18.3 and 22.1% of the women had one and more than five living children, respectively. Thirty-nine percent of the women were giving their first birth before the age of 18 years, and 79% of the women desired children more. Of the total, 26.7 and 7.4% of women delivered at the health facility and received a postnatal check with 2 months, respectively. Of those who received a postnatal check, 51.9% of them got the check provided by nurses (Table 2).

**Table 2:** Obstetric characteristics of study participants in the emerging regions of Ethiopia, EDHS 2016 (*n* = 1406)

Variables	Category	Frequency (n)	Percent (%)
ANC visit	Yes	787	56.0
	No	619	44.0
Number of ANC visits (*n* = 787)	Once	83	10.6
	Twice	122	15.5
	Three times	233	29.6
	Four times and above	349	44.3
Timing of 1st ANC check in months (*n* = 787)	1–2	79	10.1
	3–7	670	85.1
	> = 8	38	4.8
The pregnancy was wanted	Yes	1262	89.8
	No	144	10.2
Number of a living child	1	257	18.3
	2–5	838	59.6
	6+	311	22.1
Age of the mother at first birth (in years)	< 18	554	39.4
	18–24	775	55.1
	25+	77	5.5
Desire for more children	Wants	1111	79.0
	Undecided	68	4.8
	Wants no more	227	16.2
Place of delivery	Home	1031	73.3
	Health facility	375	26.7
PNC check within 2 months	Yes	104	7.4
	No	1302	92.6
Who provided PNC services? (*n* = 104)	Doctor	10	9.6
	Nurse	54	51.9
	Midwife	5	4.8
	HEWs	32	30.8
	Others	3	2.9

### Child characteristics and common childhood illness

Nearly half (53.0%) of the children included in the analysis were male, and the mean age of the child was 10.2 months (SD: 6.4). Moreover, 26.5% of the children had large birth weights, and 17.1% were in the first birth order. Nearly one-fourth (23.5%) of the women had less than 24 months of preceding birth interval. Of the total, 13.2, 12.3, and 14.1% of children had diarrhoea, cough, and fever within the last 2 weeks of the survey, respectively. For 7.3 and 14.1% of the women, the source of information about breastfeeding was TV and radio, respectively (Table 3).

**Table 3:** Child characteristics and information sources for breastfeeding among participants in the emerging regions of Ethiopia, EDHS 2016 (*n* = 1406)

Variables	Category	Frequency (n)	Percent (%)
Sex of the child	Male	746	53.0
	Female	660	47.0
Current age of the child in months	0–6	484	34.4
	7–12	381	27.1
	13–23	541	38.5
Size of the child at birth	Large	372	26.5
	Average	561	39.9
	Small	473	33.6
Birth order	1	240	17.1
	2–5	788	56.0
	6+	378	26.9
Preceding birth interval in months	<=24	331	23.5
	25–36	393	28.0
	37–48	238	16.9
	> = 49	444	31.6
Had diarrhoea recently	Yes	185	13.2
	No	1221	86.8
Had cough recently	Yes	173	12.3
	No	1233	87.7
Had fever recently	Yes	198	14.1
	No	1208	85.9

### Community-level poverty, women empowerments, and access to health services

The finding showed that 37.9% of the women were empowered, and only 2 % of the communities had media exposure. Of the total participants, 11.4% of the communities were in the rich wealth status and for 42.2% of the community’s distance to the health facilities was not a problem (Table 4).

**Table 4:** Community-level poverty, women empowerment and access to health services among participants in the emerging regions of Ethiopia, EDHS 2016 (*n* = 1406)

Variables	Response	Frequency	Percent
Women empowerment	No	837	62.1
	Yes	510	37.9
Community media exposure	No	1378	98.0
	Yes	28	2.0
Community poverty	Poor	1126	80.1
	Middle	120	8.5
	Rich	160	11.4
Distance to the nearest health facility	Not a problem	596	42.4
	Big problem	810	57.6

### Coverage of exclusive breastfeeding practices

The majority (84.3%) of the children currently feed on breast. Sixty-four percent of the children were initiated breastfeeding immediately after birth. Only 17.6% (95% CI: 15.6–19.6) of the children were on exclusive breastfeeding (Table 5).

**Table 5:** Coverage of exclusive breastfeeding among children aged 0–23 months in the emerging regions of Ethiopia, EDHS 2016 (*n* = 1406)

Variables	Category	Frequency (n)	Percent (%)
Child breastfeed	Ever breastfeed (not currently)	178	12.7
	Still breastfeeding	1185	84.3
	Never breastfeed	43	3.0
Initiation of breastfeeding (*n* = 1363)	Immediately	879	64.5
	Within an hour	44	3.2
	1–24 Hrs	288	21.1
	> 24 Hrs	152	11.2
Exclusive breastfeed	Yes	247	17.6
	No	1159	82.4

### The multilevel mixed-effect binary logistic regression analysis

The multilevel model was confirmed by intra-cluster correlation coefficient (ICC) of 45%, which means 45% of the variation in exclusive breastfeeding practices among children aged 0 to 23 months was due to the variation between clusters. Then the four models (null model, individual-level, community level, and a model with both individual and community-level factors) were compared to estimate the best fit with the lowest deviance value (Model 3) (Table 6).

**Table 6:** Model comparison for identifying factors affecting exclusive breastfeeding practices among children aged 0–23 months in the emerging regions of Ethiopia, 2016

Model	Deviance	AIC
Null model (Without independent variables)	2352.16	2357.08
Model 1 (Individual-variables)	868.58	922.59
Model 2 (Community-level variables)	1236.28	1252.28
Model 3 (Both individual and community-level variables	794.98	860.98

### Factors associated with exclusive breastfeeding practices (mixed-effects)

As shown in Table 7, maternal literacy, employment status, household wealth status, religion, ANC visits, size of the child at birth, number of living children in the household, desire for more children, history of diarrhoea, cough, and fever within the last 2 weeks were the individual level candidate variables. Whereas region, community-level poverty, and community women empowerments were the community level candidate variables.

**Table 7:** Multilevel logistic regression analysis of individual and community-level factors associated with exclusive breastfeeding practices among children aged 0–23 months in the emerging regions of Ethiopia, EDHS 2016 (*n* = 1406)

Variables	Exclusive breastfeed		COR (95%CI)	Null model (ICC = 45%)	Model 1 AOR (95% CI)	Model 2 AOR (95% CI)	Model 3 (full model) AOR (95% CI)
	Yes n	No n					
Literacy status							
Literate	46	175	1		1		1
Illiterate	201	984	0.75 (0.52–1.10)		0.76 (0.48–1.19)		0.83 (0.52–1.34)
Employment status							
Not employed	212	869	1		1		1
Employed	35	290	0.45 (0.30–0.68)		0.45 (0.29–0.67)		0.33 (0.21–0.53) *
Wealth status							
Poorest	159	711	1		1		1
Poorer	22	137	0.69 (0.42–1.13)		0.75 (0.44–1.26)		0.59 (0.33–1.05)
Middle	19	82	1.03 (0.60–1.77)		1.08 (0.60–1.94)		0.56 (0.24–1.32)
Richer	19	81	1.03 (0.59–1.78)		0.88 (0.49–1.58)		0.39 (0.16–0.96) *
Richest	28	148	0.84 (0.53–1.34)		0.65 (0.37–1.14)		0.62 (0.32–1.19)
Religion							
Muslim	166	845	1		1		1
Protestant	47	201	1.19 (0.82–1.74)		1.31 (0.86–1.99)		1.17 (0.61–2.24)
Orthodox	25	84	1.50 (0.92–2.47)		1.59 (0.92–2.76)		0.99 (0.52–1.89)
Others	9	29	1.62 (0.73–3.58)		2.09 (0.90–4.82)		1.69 (0.66–4.35)
ANC visit							
No	101	518	1		1		1
1–3	76	362	1.08 (0.77–1.51)		1.28 (0.90–1.84)		1.29 (0.90–1.86)
4+	70	279	1.27 (0.90–1.81)		1.53 (1.01–2.30)		1.31 (0.86–2.02)
Size of the child at birth							
Large	65	307	1		1		1
Average	98	481	0.97 (0.68–1.37)		1.00 (0.69–1.44)		1.05 (0.72–1.53)
Small	84	371	1.08 (0.75–1.56)		1.19 (0.81–1.76)		1.30 (0.86–1.98)
Number of living children							
One	44	197	1		1		1
Two	54	211	1.13 (0.72–1.78)		1.26 (0.78–2.02)		1.23 (0.76–2.00)
Three	33	199	0.73 (0.44–1.21)		0.83 (0.49–1.41)		0.83 (0.47–1.41)
Four	36	161	0.98 (0.60–1.62)		1.09 (0.65–1.85)		1.06 (0.62–1.82)
Five	29	120	1.06 (0.62–1.81)		1.13 (0.64–1.99)		1.06 (0.59–1.91)
Six and above	51	271	0.84 (0.53–1.32)		0.96 (0.58–1.58)		0.83 (0.49–1.40)
Desire more child							
Wants	187	924	1		1		1
Undecided	18	50	1.79 (1.01–3.19)		1.97 (1.09–3.58)		2.29 (1.21–4.29) *
Wants no more	42	185	1.09 (0.75–1.60)		1.13 (0.74–1.72)		0.90 (0.56–1.44)
Diarrhea in the last 2 weeks							
No	233	988	1		1		1
Yes	14	171	0.34 (0.19–0.60)		0.37 (0.21–0.69)		0.31 (0.16–0.61) *
Cough in the last 2 weeks							
No	231	1002	1		1		1
Yes	16	157	0.43 (0.25–0.75)		0.63 (0.33–1.17)		0.69 (0.36–1.33)
Fever in the last 2 weeks							
No	228	980	1		1		1
Yes	19	179	0.45 (0.27–0.75)		0.72 (0.39–1.32)		0.71 (0.37–1.34)
Region							
Afar	61	297	1			1	1
Somali	71	425	0.81 (0.55–1.19)			0.84 (0.56–1.26)	0.99 (0.65–1.53)
Benishangul	75	232	1.58 (1.06–2.36)			1.64 (1.06–2.52)	2.63 (1.44–4.82) *
Gambela	40	205	0.94 (0.60–1.48)			0.88 (0.54–1.43)	1.04 (0.48–2.26)
Community poverty							
Poor	192	934	1			1	1
Middle	20	100	0.96 (0.57–1.61)			0.87 (0.51–1.49)	1.31 (0.62–2.76)
Rich	35	125	1.34 (0.88–2.04)			1.15 (0.74–1.80)	2.05 (0.96–4.40)
Community women empowerment							
No	101	423	1			1	1
Yes	136	687	0.81 (0.60–1.09)			0.80 (0.59–1.07)	0.82 (0.60–1.11)

*AOR* adjusted odds ratio, *COR* crude odds ratio, *ICC* intra-class correlation coefficient, *ANC* antenatal care, *PNC* postnatal care, *Model 0* a model for the intra-class correlation coefficient (null model), *Model 1* adjusted for individual-level characteristics, *Model 2* adjusted for community-level characteristics, *Model 3* adjusted for both individual and community-level characteristics (full model)

*statistically significant at *p*-value < 0.05

After adjusting for individual and community-level factors (model 3), women’s employment status, household wealth status, desire for more children, history of diarrhoea in the last 2 weeks, and region were significantly associated with the low coverage of exclusive breastfeeding for less than 6 months among children aged under 23 months.

Accordingly, children whose mothers/caregivers were employed were 67.0% less likely to receive exclusive breastfeeding compared to those children whose mothers/caregivers were unemployed (AOR: 0.33, 95% CI: 0.21–0.53). Children born from richer mothers were 61% less likely to practice exclusive breastfeeding compared to those who were born from the poorest mothers (AOR: 0.39, 95% CI: 0.16–0.96). Those mothers who were undecided for having more children were 2.29 times more likely to practice exclusive breastfeeding compared to those who want more children (AOR: 2.29, 95% CI: 1.21–4.29). Those children who had a history of diarrhoea in the last 2 weeks were 69.0% less likely to feed on breast exclusively compared to their counterparts (AOR: 0.31, 95% CI: 0.16–0.61). Moreover, children who live in the Benishangul region were 2.63 times more likely to receive exclusive breastfeeding compared to those children who live in the Afar region (AOR: 2.63, 95% CI: 1.44–4.82).

## Discussion

The study revealed that the magnitude of exclusive breastfeeding among children under 23 months in the emerging regions of Ethiopia was 17.6%. The finding is comparable with that of a study carried out in Nigeria (19%) [7]. However, it is lower than those of studies conducted in Somali Land (20.47%) [36], Fanfan Zone (24.8%) [27], Addis Ababa, Ethiopia (29.3%) [21], Gondar, Ethiopia (34.8%) [50], India (48.6%) [51], Amhara regional state, Ethiopia (50.1%) [52], Jigjiga, Ethiopia (54.91%) [26], Tanzania (59%) [29], Bahirdar, Ethiopia (59.7%) [24], Goba District, Ethiopia (71.3%) [53], Southern Ethiopia (78%) [23], Ghana (64%) [54], Debrebirhan, Ethiopia (68.6%) [55], and Azezo District, Ethiopia (79%) [22]. The difference might be attributed to numerous possible reasons which were explained in different studies. The possible reason might be due to the differences in community awareness and access to information about EBF, access to health facilities, provision of ANC and PNC services, cross-cultural differences [4, 22, 24] and socio-economic characteristics. In this regard, emerging regions in Ethiopia had distinct geographic, demographic, and economic characteristics. They were victims of past development policies [44], which resulted in these regions to have the inaccessibility of information and health facility as well as different health services that lead to low EBF practices. These emerging regions have dry weather conditions that urge the mother to give water, as a mistaken belief that water is needed even if it is not scientifically recommended and not needed during exclusive breastfeeding, for their infants to wet their mouth or to satisfy their thirst [54, 56]. Due to the absence of enough rain in these emerging regions, enough food realising crops will not be produced; which resulted in the mother not to get a balanced diet. Therefore, the mothers will not produce enough breast milk for practising EBF [29].

On the other hand, the finding of our study is higher than studies carried out in Djibouti (1%) [57] and Kenya (12.6%) [38]. The possible justification for this difference between our study and Djibouti could be due to Djibouti’s hot weather that causes the infant often feels thirsty which forced the mother to give water, as a mistaken belief that water is needed even if it is not needed during exclusive breastfeeding [57] and that leads to low EBF practices. Besides, the other explanation for the difference between our study and the one which conducted in Kenya might be cultural and study time variations of policy establishment, advocacy, and implementation. This can be elaborated; there is a five-year difference between our study and Kenya’s study, which would have a significant effect on the advocacy and policy agenda about the importance of EBF.

In this study, women’s employment status, household wealth status, desire for more children, history of diarrhoea in the last 2 weeks, and region were significantly associated with the practices of exclusive breastfeeding for less than 6 months among mothers who had children aged under 23 months. Accordingly, children who have employed mothers/caregivers were less likely to receive exclusive breastfeeding than those children whose mothers/caregivers were not employed. This finding is comparable with other studies carried out both in Ethiopia [22, 24, 27, 50, 52, 53, 55] and elsewhere [7, 58, 59]. The possible reason might be because employed mothers might return to their work within a short period after giving birth and resulted in the mothers to have high workload as well as short rest time [7, 27, 50, 55]. Therefore, they could not have enough time to practice and maintain EBF. Moreover, the other possible explanation might be if the mothers work outside their home, then their workplace could be distant from their home, near-site child care centres would be absent, which leads them not to access private space in the workplace for practising EBF [19, 55]. As a result, exclusive breastfeeding practice might be lower among employed mothers.

Children born from richer mothers had lower exclusive breastfeeding practice compared to those who were born from the poorest mothers. This finding is in line with studies conducted in Ethiopia [21, 27, 52] and India [51]. It could be attributed to the mothers who have lower household income might not have any option other than breast milk to supply an additional food source for their children [27, 52]. On the other hand, high household income would be related to greater affordability of foods for infant other than breast milk [51] because of wealthier mothers can afford breast milk substitutes or status foods. Therefore, mothers who have a lower household income might have high exclusive breastfeeding practice. Whereas, the finding of this study regarding household wealth status /income/ is in contrast with other studies conducted in Azezo district, Ethiopia [22] and Somali Land [36].

This study revealed that mothers who had undecided for having more children were highly practised exclusive breastfeeding compared to those who wanted/decided to have more children. This finding is supported by the previous study conducted in Ethiopia [30]. The possible justification might be if the mothers have a desire to have more children, they might get pregnant within a short period after giving birth. In this regard, some mothers in our country assume that if an infant obtains breast milk from a pregnant mother, the infant might experience diarrhoea and weight loss. Hence, to control such diarrhoea and weight loss, the mothers who desire to have more children might start complementary feeding that leads to low exclusive breastfeeding practice.

In this study, children who had a history of diarrhoea in the last 2 weeks were less likely to practice exclusive breastfeeding compared to their counterparts. Studies conducted in Nigeria [42] and a systematic review carried out in European countries [60] showed the association between exclusive breastfeeding and diarrhoea. Surprisingly, the presence of diarrhoea could be the effect of this poor exclusive breastfeeding practice, and this is strongly supported by a systematic review carried out in European countries which estimated that promotion of exclusive breastfeeding for less than 6 months age group infants would reduce diarrhoea by 8 to 20% [60]. Consistent with this finding, another study also scientifically argued that the relationship between diarrhoea and EBF was due to lactoferrin protein, which is found in every human breast milk who have a child of all age group. Likewise, this lactoferrin which is a growth factor of lymphocytes, can destroy disease-causing pathogens to reduce the inflammatory response and can improve the activity of the immune system [61].

Moreover, children who lived in the Benishangul region received more exclusive breastfeeding compared to those children who live in the Afar region. This is in agreement with a previous study done in Ethiopia [19]. The possible explanation for this could be traditional practices, way of life, and cultural belief differences of the population lived in these regions [19, 25, 54]. For example, one previous study conducted in the Afar region identified a traditional practice was giving raw cow or goat butter, milk, and or water for newly born neonates to be swallow immediately after birth as a culture to benefit their child [25]. Hence, the traditions mentioned above of this region might negatively affect EBF practices. Furthermore, differences inaccessibility of information, healthcare facilities, and healthcare services due to the lag behind in infrastructural developments, drought, and poverty might be the other mentioned reasons [4, 44].

### Strength and limitation of the study

The major strengths of this study are; it showed the actual exclusive breastfeeding practices among in the pastoral communities of the country using the national representative and large sample size, which could be generalised to other similar settings. Besides, this study used a multilevel-modelling technique, which takes into consideration the hierarchical nature of the survey data to identify the individual and community-level factors.

The study tried to measure the exclusive breastfeeding among children aged 0–23 months; however, mothers might experience recall bias for those children aged higher than 12 months, to recollect their first 6 months feeding practices. Moreover, EBF was measured for 0–6 months and 7–16 months and finally merged, which might affect the overall measurements. The other possible bias might be the social desirability bias because the data collectors were health professionals.

## Conclusions

Exclusive breastfeeding practices in the emerging regions of Ethiopia was surprisingly low. Mothers who are employed, being richer, and child with a history of diarrhoea were factors that affected the practices negatively. In contrast, mothers who have undecided to have more children and those who live in the Benishangul region were relatively positive contributor for exclusive breastfeeding practices. Therefore, healthcare workers and policymakers should do more to increase exclusive breastfeeding practices in the regions.

## Data Availability

The data used for the current study will be available upon reasonable requests from the corresponding author.

## Abbreviations

AIC: Akaike’s Information Criterion
ANC: Antenatal care
AOR: Adjusted odds ratio
COR: Crude odds ratio
CSA: Central Statistical Agency
EA: Enumeration area
EBF: Exclusive breastfeeding
EDHS: Ethiopian Demographic and Health Survey
ICC: Intra-class correlation coefficient
PNC: Postnatal care
WHO: World Health Organization

## References

[1] Geneva W: Indicators for assessing breast feeding practices. WHO Geneva, Switzerland: WHO Document WHO/CDD/SER 1991, 91:14.

[2] Ladomenou F, Moschandreas J, Kafatos A, Tselentis Y, Galanakis E (2010). Protective effect of exclusive breastfeeding against infections during infancy: a prospective study. Arch Dis Child.

[3] Gibson RS, Abebe Y, Hambidge KM, Arbide I, Teshome A, Stoecker BJ (2009). Inadequate feeding practices and impaired growth among children from subsistence farming households in Sidama, southern Ethiopia. Matern Child Nutr.

[4] Lauer JA, Betrán AP, Barros AJD, de Onís M (2006). Deaths and years of life lost due to suboptimal breastfeeding among children in the developing world: a global ecological risk assessment. Public Health Nutr.

[5] Anatolitou F (2012). Human milk benefits and breastfeeding. J Pediatr Neonatal Individualized Med.

[6] Bosnjak A, Grgurić J (2007). Long-term health effects of breastfeeding. Lijecnicki vjesnik.

[7] Agunbiade OM, Ogunleye OV (2012). Constraints to exclusive breastfeeding practice among breastfeeding mothers in Southwest Nigeria: implications for scaling up. Int Breastfeed J.

[8] UNICEF.: Progress for children: a report card on nutrition: Unicef; 2006.

[9] Organisation WH (2002). Infant and young child nutrition: global strategy on infant and young child feeding. Report by the Secretariat.

[10] Roberts T, Carnahan E, Gakidou E (2013). Burden attributable to suboptimal breastfeeding: a cross-country analysis of country-specific trends and their relation to child health inequalities. Lancet.

[11] Scherbaum V, Srour ML: The role of breastfeeding in the prevention of childhood malnutrition. In: Hidden Hunger. Volume 115, edn.: Karger Publishers; 2016: 82–97. 10.1159/000442075 27198529

[12] Kneepkens CF, Brand PL (2010). Clinical practice: breastfeeding and the prevention of allergy. Eur J Pediatr.

[13] Ehlayel MS, Bener A: Duration of breastfeeding and the risk of childhood allergic diseases in a developing country. In: Allergy and asthma proceedings: 2008: OceanSide Publications; 2008: 386. 10.2500/aap.2008.29.3138 18702886

[14] Bhandari N, Kabir AI, Salam MA (2008). Mainstreaming nutrition into maternal and child health programmes: scaling up of exclusive breastfeeding. Matern Child Nutr.

[15] Bener A, Ehlayel M, Alsowaidi S, Sabbah A (2007). Role of breast feeding in primary prevention of asthma and allergic diseases in a traditional society. Eur Ann Allergy Clin Immunol.

[16] Grummer-Strawn LM, Mei Z (2004). Does breastfeeding protect against pediatric overweight? Analysis of longitudinal data from the Centers for Disease Control and Prevention pediatric nutrition surveillance system. Pediatrics.

[17] Davis MK (2001). Breastfeeding and chronic disease in childhood and adolescence. Pediatr Clin N Am.

[18] Butte NF, Lopez-Alarcon MG, Garza C: Nutrient adequacy of exclusive breastfeeding for the term infant during the first six months of life: World Health Organization; 2002.

[19] Ahmed KY, Page A, Arora A, Ogbo FA (2019). Trends and determinants of early initiation of breastfeeding and exclusive breastfeeding in Ethiopia from 2000 to 2016. Int Breastfeed J.

[20] Central Statistical Agency (CSA) [Ethiopia] and ICF (2016). Ethiopia Demographic and Health Survey 2016.

[21] Shifraw T, Worku A, Berhane Y (2015). Factors associated exclusive breastfeeding practices of urban women in Addis Ababa public health centers, Ethiopia: a cross sectional study. Int Breastfeed J.

[22] Asemahagn MA (2016). Determinants of exclusive breastfeeding practices among mothers in azezo district, Northwest Ethiopia. Int Breastfeed J.

[23] Lenja A, Demissie T, Yohannes B, Yohannis M (2016). Determinants of exclusive breastfeeding practice to infants aged less than six months in Offa district, southern Ethiopia: a cross-sectional study. Int Breastfeed J.

[24] Seid AM, Yesuf ME, Koye DN (2013). Prevalence of exclusive breastfeeding practices and associated factors among mothers in Bahir Dar city, Northwest Ethiopia: a community based cross-sectional study. Int Breastfeed J.

[25] Tsegaye M, Ajema D, Shiferaw S, Yirgu R (2019). Level of exclusive breastfeeding practice in remote and pastoralist community, Aysaita woreda, Afar, Ethiopia. Int Breastfeed J.

[26] Obsiye M (2017). Assesing the association between infant formula Promoion and exclusive breast feeding practice among mothers of infants aged 0–5 months.

[27] Tadesse F, Alemayehu Y, Shine S, Asresahegn H, Tadesse T (2019). Exclusive breastfeeding and maternal employment among mothers of infants from three to five months old in the Fafan zone, Somali regional state of Ethiopia: a comparative cross-sectional study. BMC Public Health.

[28] Rollins NC, Bhandari N, Hajeebhoy N, Horton S, Lutter CK, Martines JC, Piwaz E, Richter LM, Victora CG (2016). Breastfeeding 2: why invest, and what it will take to improve breastfeeding practices. Lancet.

[29] Dede KS, Bras H (2020). Exclusive breastfeeding patterns in Tanzania: do individual, household, or community factors matter?. Int Breastfeed J.

[30] Woldeamanuel BT (2020). Trends and factors associated to early initiation of breastfeeding, exclusive breastfeeding and duration of breastfeeding in Ethiopia: evidence from the Ethiopia demographic and health survey 2016. Int Breastfeed J.

[31] Kebede T, Woldemichael K, Jarso H, Bekele BB (2020). Exclusive breastfeeding cessation and associated factors among employed mothers in Dukem town, Central Ethiopia. Int Breastfeeding J.

[32] Talbert A, Jones C, Mataza C, Berkley JA, Mwangome M (2020). Exclusive breastfeeding in first-time mothers in rural Kenya: a longitudinal observational study of feeding patterns in the first six months of life. Int Breastfeed J.

[33] Gebrekidan K, Fooladi E, Plummer V, Hall H (2020). Enablers and barriers of exclusive breastfeeding among employed women in low and lower middle-income countries. Sex Reprod Healthcare.

[34] Alebel A, Tesma C, Temesgen B, Ferede A, Kibret GD (2018). Exclusive breastfeeding practice in Ethiopia and its association with antenatal care and institutional delivery: a systematic review and meta-analysis. Int Breastfeed J.

[35] Egata G, Berhane Y, Worku A (2013). Predictors of non-exclusive breastfeeding at 6 months among rural mothers in East Ethiopia: a community-based analytical cross-sectional study. Int Breastfeed J.

[36] Jama A, Gebreyesus H, Wubayehu T, Gebregyorgis T, Teweldemedhin M, Berhe T, Berhe N (2020). Exclusive breastfeeding for the first six months of life and its associated factors among children age 6-24 months in Burao district, Somaliland. Int Breastfeed J.

[37] Kumar A, Singh V (2015). A study of exclusive breastfeeding and its impact on nutritional status of child in EAG states. J Stat Appl Probability.

[38] Muchina E, Waithaka P: Relationship between breastfeeding practices and nutritional status of children aged 0–24 months in Nairobi, Kenya, Afr J Food Agric Nutr Dev. 2010;10(4).

[39] Lamberti LM, Walker CLF, Noiman A, Victora C, Black RE (2011). Breastfeeding and the risk for diarrhea morbidity and mortality. BMC Public Health.

[40] Bartick MC, Stuebe AM, Schwarz EB, Luongo C, Reinhold AG, Foster EM (2013). Cost analysis of maternal disease associated with suboptimal breastfeeding. Obstet Gynecol.

[41] Roberts TJ, Carnahan E, Gakidou E (2013). Can breastfeeding promote child health equity? A comprehensive analysis of breastfeeding patterns across the developing world and what we can learn from them. BMC Med.

[42] Ogbo FA, Okoro A, Olusanya BO, Olusanya J, Ifegwu IK, Awosemo AO, Ogeleka P, Page A (2019). Diarrhoea deaths and disability-adjusted life years attributable to suboptimal breastfeeding practices in Nigeria: findings from the global burden of disease study 2016. Int Breastfeed J.

[43] Unar-Munguía M, Meza R, Colchero MA, Torres-Mejía G, de Cosío TG (2017). Economic and disease burden of breast cancer associated with suboptimal breastfeeding practices in Mexico. Cancer Causes Control.

[44] Gebre-Egziabhere T (2018). Emerging regions in Ethiopia: are they catching up with the rest of Ethiopia?. East Afr Soc Sci Res Rev.

[45] Stark J, Terasawa K, Ejigu M. Climate change and conflict in pastoralist regions of Ethiopia: mounting challenges, emerging responses. Conflict Management and Mitigation (CMM) Discussion Paper 2011(4).

[46] Organization WH (2008). Indicators for assessing infant and young child feeding practices: part 1: definitions: conclusions of a consensus meeting held 6–8 November 2007 in Washington DC, USA.

[47] Organization WH (2003). Complementary feeding: report of the global consultation, and summary of guiding principles for complementary feeding of the breastfed child.

[48] Organisation WH, UNICEF (2002). Global Strategy for Infant and Young Child Feeding (endorsed by the 55th World Health Assembly meeting in its resolution WHA 55/. 25 May 2002).

[49] Hox JJ, Moerbeek M, Van de Schoot R: Multilevel analysis: techniques and applications, 3: Routledge; 2010.

[50] Chekol DA, Biks GA, Gelaw YA, Melsew YA (2017). Exclusive breastfeeding and mothers’ employment status in Gondar town, Northwest Ethiopia: a comparative cross-sectional study. Int Breastfeed J.

[51] Chandhiok N, Singh KJ, Sahu D, Singh L, Pandey A (2015). Changes in exclusive breastfeeding practices and its determinants in India, 1992–2006: analysis of national survey data. Int Breastfeed J.

[52] Tewabe T, Mandesh A, Gualu T, Alem G, Mekuria G, Zeleke H (2016). Exclusive breastfeeding practice and associated factors among mothers in Motta town, east Gojjam zone, Amhara regional state, Ethiopia, 2015: a cross-sectional study. Int Breastfeed J.

[53] Setegn T, Belachew T, Gerbaba M, Deribe K, Deribew A, Biadgilign S (2012). Factors associated with exclusive breastfeeding practices among mothers in Goba district, south East Ethiopia: a cross-sectional study. Int Breastfeed J.

[54] Tampah-Naah AM, Kumi-Kyereme A (2013). Determinants of exclusive breastfeeding among mothers in Ghana: a cross-sectional study. Int Breastfeed J.

[55] Asfaw MM, Argaw MD, Kefene ZK (2015). Factors associated with exclusive breastfeeding practices in Debre Berhan District, Central Ethiopia: a cross sectional community based study. Int Breastfeed J.

[56] Fjeld E, Siziya S, Katepa-Bwalya M, Kankasa C, Moland KM, Tylleskär T, Group P-ES (2008). ‘No sister, the breast alone is not enough for my baby’a qualitative assessment of potentials and barriers in the promotion of exclusive breastfeeding in southern Zambia. Int Breastfeed J.

[57] Bureau WR, Headquarters W (2011). WFP Djibouti.

[58] El-Gilany A-H, Shady E, Helal R (2011). Exclusive breastfeeding in Al-Hassa, Saudi Arabia. Breastfeed Med.

[59] Dearden K, Altaye M, Maza ID, Oliva MD, Stone-Jimenez M, Morrow AL, Burkhalte BR (2002). Determinants of optimal breast-feeding in peri-urban Guatemala City, Guatemala. Rev Panam Salud Publica.

[60] Organisation WH, Horta B, Victora C: A systematic review on the benefits of breastfeeding on diarrhoea and pneumonia mortality. 2013. World Health Organization.

[61] Hashizume S, Kuroda K, Murakami H (1983). Identification of lactoferrin as an essential growth factor for human lymphocytic cell lines in serum-free medium. Biochim Biophys Acta (BBA)-Mol Cell Res.

